# The development of L2 collocational familiarity and its relationship with collocational frequency and congruency

**DOI:** 10.3389/fpsyg.2024.1332692

**Published:** 2024-07-01

**Authors:** Jie Lou

**Affiliations:** School of Humanities and Foreign Language, Zhejiang Shuren University, Hangzhou, China

**Keywords:** L2 collocational familiarity, collocational frequency, congruency, L2 proficiency, relationship

## Abstract

The present study took L2 English learners of different levels in China as subjects to investigate the relationship between collocational familiarity and collocational frequency as well as L1-L2 congruency, and then explored the development of the above relationship as L2 proficiency develops. The results showed that: a moderate positive correlation existed between familiarity and frequency, and the correlation increased with proficiency; a moderate positive correlation also existed between familiarity and congruency, but the correlation decreased with proficiency. Based on previous studies and the present findings, the research group infer that: low familiarity collocations tend to be represented and processed in analytic way and same-translation effect helps accelerate the semantic access of congruent collocations in this process; with the increase in learners’ proficiency, collocation familiarity develops from low to high with frequency effect; high familiarity collocations tend to be represented and processed in holistic way to have direct semantic access; furthermore, learners of two levels have two kinds of collocational representation and semantic access, but low-level learners show more analytic representation and indirect semantic access because of having more low familiarity collocations in the mental lexicon.

## 1 Introduction

As a typical form of formulaic language, collocations exist widely in almost all the languages. Existing research has shown that collocations are not only crucial for L1 acquisition but also essential for L2 learners to fluently use the target language (Wray, 2002). Wang (2019, p. 74) regarded collocations as a construction and believed that they were the foundation for natural, authentic, fluent, and efficient language use, as well as the source of language productivity, making it of utmost importance in second language learning. The past decade witnessed an increased interest in L2 collocation research (Yamashita and Jiang, 2010; Wolter and Gyllstad, 2011; Sonbul, 2015; Zhang et al., 2017, 2021; Cai and Xu, 2018; Zhang and Fang, 2020). From the perspective of psycholinguistics, research on L2 collocations focuses on its processing mechanisms, mainly including the L2 collocation representation and the factors influencing collocation processing, such as collocational frequency, L1-L2 congruency, transparency, and contextual strength. Among them, collocational frequency and congruency have been proven to be important influencing factors (Wolter and Gyllstad, 2011, 2013; Wang, 2015; Wei and Zhang, 2017; Wu and Lou, 2017; Zhang et al., 2017; Lou, 2022a,b).

However, the above factors are mostly objective variables, and few scholars have studied collocations from the perspective of familiarity which is a subjective variable. According to existing empirical studies (Wei and Zhang, 2017; Chen et al., 2020), vocabulary familiarity is an important factor affecting L2 vocabulary processing. The higher the vocabulary familiarity is, the faster the language is processed. Furthermore, Jiang (2018) also pointed out that observed frequency effect could be actually a familiarity effect or a mixture of two.

Vocabulary familiarity includes word familiarity and multi-word unit familiarity, such as collocational familiarity. Word familiarity is viewed as a property of the representations stored in memory, in particular words or as a state of processing (Cordier and Jean-Francois Le, 2005, p. 528). However, collocational familiarity is different from word familiarity because collocations have a certain level of semantic opacity, which means that collocation meaning is not simply the sum of component words meaning (e.g., soft opening). Collocational familiarity could be influenced by word familiarity, but the operational definition of collocational familiarity in this paper is from receptive dimension: the extent for learners to recognize the overall semantic meaning of collocations based on familiar component words, with subjective ratings ranging from “unfamiliar” to “familiar.” If learners can easily associate a collocation form with its overall meaning, the collocation is considered to have high familiarity, otherwise it has low familiarity. Therefore, although collocational familiarity is a subjective judgment of learners, it reflects the psychological representation of learners’ collocation memory. So far, the existing research on vocabulary familiarity mainly stays at the word level (e.g., Cordier and Jean-Francois Le, 2005; Tanaka-Ishii and Terada, 2011; Zhang and Wang, 2012), and there is a scarcity of investigation on the familiarity of multi-word units including collocations.

Familiarity is closely related to frequency. Sun and Li (2017) argued that high frequency was a necessary condition for L2 vocabulary acquisition. As the main factor influencing collocation processing, collocational frequency is a kind of native language frequency. It is based on the co-occurrence frequency of component words in language data, which is statistically derived from L1 corpora such as the British National Corpus (BNC) and Corpus of Contemporary American English (COCA) created by Davies (2008). L2 collocational familiarity reflects the input frequency of collocations in a second language environment. Learners are more familiar with collocations that have a higher input frequency, and vice versa. Thus, the comparison between familiarity and collocation frequency is actually a comparison between L2 frequency and frequency of native speakers. Relevant studies have shown that these two types of frequencies are correlated to a certain extent. For example, Siyanova and Schmitt (2008) compared the intuitions of native speakers and L2 learners regarding collocational frequency, and found that although the intuitions L2 learners of collocational frequency were less accurate than those of native speakers, they could still distinguish between high and low frequency collocations. Wu and Lou (2017) compared the intuitions of collocational frequency of L2 English learners in China with BNC frequency data, and found a moderate correlation between the two. Additionally, in Wu and Lou’s (2017) study, high-level learners showed slightly better accuracy in judging low-frequency collocations compared to low-level learners. Although the above studies do not focus on familiarity, they all indicate that L2 learners are generally sensitive to native language frequency. Based on this, the research group hypothesize that there is also a certain correlation between L2 collocational familiarity and native collocational frequency. This study will compare and analyze collocational familiarity and native collocational frequency to verify the above hypothesis, and at the same time, explore the changes in L2 collocation representation as their proficiency improves.

Another important factor influencing familiarity with L2 collocations is L1-L2 congruency. This is because L2 learning is inevitably influenced by learners’ native language. Congruent collocations are those that, “when translated word for word, are felicitous in both the first language (L1) and the L2” (Wolter and Yamashita, 2015, p. 1195). Thus L2 collocations can be roughly divided into congruent collocations and non-congruent collocations. Research by Yamashita and Jiang (2010), Wolter and Gyllstad (2011), and Zhang et al. (2017) showed that the L1 medium was an objective existence, and the native language played an accelerating effect in the processing of congruent L2 collocations, known as the “same-translation effect.” Few studies demonstrated that the familiarity of collocations played a moderating role in the processing of collocations with different degrees of congruency. For example, Chen et al. (2016) and Wei and Zhang (2017) both showed in their research that only if the degree of collocation familiarity was low, the reaction time used in recognizing congruent collocates was significantly shorter than that used in recognizing non-⁃congruent collocates. However, these studies did not differentiate the learners’ proficiency levels, so they could not confirm the developmental changes in collocation representation and processing.

Furthermore, collocational familiarity, collocational frequency, and congruency may play different roles in L2 collocation processing and development. Wray (2002) pointed out that native language collocation processing was holistic representation, while L2 collocations were not. Later studies (such as Wolter and Gyllstad, 2011) provided empirical evidence to show that high-level learners’ L2 collocations could also have a holistic representation. Recent research (e.g., Wang, 2015) further suggested that even intermediate and low-level learners could have psychological reality on collocation representations. Therefore, there is a need for further investigation into the relationships between collocational familiarity and collocational frequency, congruency, as well as their development trends with learners’ proficiency improvement. Clarifying the relationships and variations among these factors can help us understand the nature and developmental patterns of L2 collocational familiarity and gain further insights into the underlying mechanisms of L2 collocation processing. This study aims to expand previous research by focusing on L2 English learners in China and conducting two experiments to explore the aforementioned questions. The specific research questions are as follows:

1. What is the relationship between collocational familiarity and collocational frequency at the same level of L2 proficiency? How does this relationship develop as L2 proficiency improves?
2. What is the relationship between collocational familiarity and congruency at the same level of L2 proficiency? How does this relationship develop as L2 proficiency improves?
3. What is the possible model of L2 collocation learning based on the relationship of collational familiarity with collocational frequency and congruency?

## 2 Experiment 1

### 2.1 Purpose of the study

The purpose of experiment 1 was to investigate the relationship between collocational familiarity and collocational frequency as well as its developmental trend.

### 2.2 Research method

#### 2.2.1 Subjects

A total of 66 native Chinese speakers students who were sophomore students from Zhejiang Shuren university in China and learned English as their second language participated in the study. Based on the Oxford Quick Placement Test scores, 31 participants with scores above the average (38.45) were categorized into the high-level English group, while the remaining 35 participants with scores below the average were categorized into the low-level English group. There was a significant difference in the average scores between the two groups (Mean *L* = 32.40(3.79), Mean *H* = 47.58(5.33), t (64) = 13.45, df = 64, *p* < 0.001). Additionally, all students received English education in Chinese schools and did not have any experience studying abroad.

#### 2.2.2 Experimental materials and the procedure

The collocations used in this experiment were selected from the BNC. To establish a uniform standard, this study only selected V + N verb collocations (e.g., make sense) or A + N noun collocations (e.g., living standard) composed of two content words, which also met the two criteria proposed by Siyanova and Schmitt (2008) for collocation definition: BNC frequency ≥ 21 and mutual information (MI) ≥ 3. According to these criteria, a total of 243 pairs of collocations were randomly sampled for this study. Based on the research findings of Fernández and Schmitt (2015), there was no significant correlation between MI and L2 collocation knowledge(*r* = 0.16). Therefore, MI was not considered as a control variable in this study. However, in order to control the influence of word frequency, word length, and congruency, the selected collocations also needed to meet the following conditions: (1) both component words were high-frequency words familiar to learners, i.e., the constituent words fall within the top 3,000 high-frequency words in the BNC (or occur more than 20,000 times per million words), (2) the word length was within 7 letters, and (3) the collocations were non-congruent evaluated by three linguistic professionals. A total of 176 collocations met these conditions. Subsequently, the research team divided them into 4 levels based on the collocational frequency in the BNC. Collocational frequencies of 21–30 were classified as low frequency, 31–60 as medium-low frequency, 61–100 as medium-high frequency, and above 100 as high frequency. From each of these frequency ranges, the research team randomly selected 10 collocations that met the criteria (5 verb collocations and 5 noun collocations), resulting in a total of 40 collocations (see Table 1). Analysis of variance showed no significant differences in average word length (*F* (3.36) = 0.007; *p* > 0.05), average word frequency (*F* (3.36) = 0, *p* > 0.05), and congruency (*F* (3.36) = 1.50, *p* > 0.05) among the four levels of collocations.

**Table 1:** Examples of collocations in frequency range.

Frequency range	Examples	Average word frequency (SD)	Average word length (SD)	Average congruency (SD)
High (10)	Make sense, flat race	39,432 (5,033)	3.56 (1.10)	1.23 (0.42)
Medium-high(10)	Bear fruit, blind date	38,435 (4,037)	4.23 (1.23)	1.20 (0.32)
Medium-low(10)	Save face, fixed sum	40,466 (5,847)	3.78 (1.23)	1.00 (0.23)
Low(10)	Reach decisions, white lie	37,293 (6,454)	4.11 (1.56)	1.12 (0.12)

To test the learners’ familiarity with these collocations, a collocational familiarity questionnaire was designed by rearranging the order of the above 40 collocation sets. The questionnaire was divided into 5 levels: level 1 corresponded to “I am not familiar at all because I completely do not know the collocation meaning,” level 2 corresponded to “I am not familiar because I do not know the collocation meaning,” level 3 corresponds to “I am not sure about the collocation meaning,” level 4 corresponded to “I am familiar because I know the collocation meaning,” level 5 corresponded to “I am very familiar because I definitely know the collocation meaning.” Participants independently completed the familiarity questionnaire within a specified time frame. To make the frequency data closer or equal to normal distribution, the original data was log-transformed before data analysis (see Appendix). After that the researchers input the frequency and familiarity variable of each collocation, as well as the participants’ L2 proficiency variable, into the computer and performed data analysis using SPSS 19.0 software.

### 2.3 Results

The research group first analyzed the correlation between collocational familiarity and collocational frequency. Considering individual collocation frequencies were excessively high (such as “take place” 10,434 times, “make sense” 1700 times), the data did not follow a normal distribution, so non-parametric analysis was used in this study. The results of Spearman correlation coefficient are shown in Table 2: the overall correlation between learners’ collocational familiarity and collocational frequency reached a moderate level and achieved significance (*r* = 0.442; *p* < 0.01); both high-level and low-level learners’ familiarity with collocations and collocation frequencies were also significantly correlated, with the correlation coefficient slightly higher for high-level learners than for low-level learners.

**Table 2:** Correlation between L2 collocational familiarity and collocational frequency of different L2 learners.

	Collocational familiarity		
	Overall	Low-level learners	High-level learners
Collocational frequency	*r* = 0.442	*r* = 0.486	*r* = 0.502
	Sig.(2-tailed) = 0.000	Sig.(2-tailed) = 0.000	Sig.(2-tailed) = 0.000

In order to fully understand the differences in familiarity among different frequency ranges and among learners of different levels, the research group subsequently conducted a stepped arrangement and comparison of familiarity data according to four frequency ranges and two level categories. The comparison results (see Table 3) showed that: (1) there were certain differences in the familiarity of both types of learners among different frequency distributions of collocations. The higher the collocation frequency was, the higher the corresponding familiarity of the learners were. Friedman’s test showed that this difference reached a statistically significant level (*p* < 0.01); (2) in the same frequency range of collocations, there were differences between learners of different levels. The familiarity of high-level learners was higher than that of low-level learners; Mann–Whitney test showed that the differences between learners also reached a statistically significant level (*p* < 0.01).

**Table 3:** L2 collocational familiarity of different learners among different frequency ranges.

	low-level learners (SD)	high-level learners (SD)	Mann-Whitney U*	p*
High frequency	3.9400 (0.73)	4.6625 (0.39)	178	0.000
Medium-high frequency	3.6086 (0.70)	4.1125 (0.71)	300	0.002
Medium-low frequency	3.3486 (0.71)	3.9469 (0.78)	282	0.001
Low frequency	2.8514 (0.51)	3.1062 (0.78)	401	0.002
Chi-Square**	61.292	59.629		
df**	3	3		
p**	0.000	0.000		

*Mann–Whitney Test; **Friedman Test.

### 2.4 Discussion

The results of Experiment 1 indicate that L2 collocational familiarity and collocational frequency are moderately positively correlated at the same proficiency level, with learners being more familiar with collocations that have a higher frequency. This conclusion is consistent with the findings of Wang (2015), as well as the survey results of Sonbul (2015) and Wu and Lou (2017), further demonstrating that L2 English learners are generally sensitive to collocational frequency. The moderate positive correlation between familiarity and frequency indicates that the overall input of collocation constructions in the second language context for the learners conforms to the distribution of “typical members with high frequency repetition and atypical members with low frequency presentation” (Bybee, 2008:233), and it produces a frequency effect. Based on the usage-based linguistics, frequency is a determining factor in language processing. Gries and Ellis (2015) pointed out that frequency determined the degree of entrenchment of constructions stored in the mental lexicon and the degree of automation of constructions in the extraction process. The research group believe that familiarity is an external manifestation of the degree of entrenchment of collocations in the mental lexicon. Repeated exposure leaves traces of low-familiarity collocations in memory, and the strength of these traces develops with increased use of collocations, gradually solidifying into holistic representations and becoming high-familiar collocations. Therefore, the higher the input frequency of typical members of collocations in the native language, the higher the corresponding degree of L2 mental entrenchment and familiarity. However, the moderate correlation (*r* = 0.442) between collocation familiarity and frequency in this study also reflects some differences between the two. This difference reflects the input differences between the native language and the second language environment. In other words, the repetition frequency of some typical members of collocations in the second language environment is not sufficient to allow learners to form holistic mental representations.

On the other hand, looking at the development trend of learners’ correlation between familiarity and frequency, the correlation coefficient of high-level learners was higher than that of low-level learners. This indicates that high-level learners are more sensitive to frequency than low-level learners. The difference in sensitivity to frequency among different learners is likely to be due to the frequency effect of language. Durrant and Schmitt (2010, p. 182) pointed out that “any deficit in collocation knowledge is a result of insufficient exposure to language.” High-level learners are more sensitive to frequency, not only because they have advantages in memory and learning methods, but also because they have had more exposure to target collocations, resulting in more high-familiar collocations. This is not to say that low-level learners cannot form holistic collocation representations. But compared with low-level learners, high-level learners may have more collocations forming holistic representations, and the degree of entrenchment is also higher. The results of this study confirm and revise Wray’s (2002) hypothesis of formulaic language, indicating that both high and low-level L2 learners have mental representations of collocations, and that the number and degree of these representations will increase synchronously with proficiency improvement.

## 3 Experiment 2

### 3.1 Purpose of the study

The purpose of experiment 2 was to investigate the relationship between L2 collocational familiarity and congruency as well as its developmental trend.

### 3.2 Research method

#### 3.2.1 Subjects

The same as those in experiment 1.

#### 3.2.2 Experimental materials and procedure

The selection criteria and range of materials in this experiment were the same as Experiment 1, but the selected items were not repeated. In addition, the purpose of this experiment was to investigate the relationship between learners’ L2 collocational familiarity and congruency. Therefore, the experimental materials controlled for frequency and other variables. The collocations must meet the following criteria: (1) the frequency and length of component words were similar to Experiment 1, within the top 3,000 highest frequency words in the BNC (or occurring more than 20,000 times per million words), and the length of the words was within 7 letters; (2) collocational frequencies were all more than 100 times in the BNC.

According to the degree of L2(English)-L1(Chinese) congruency, the research group sets three levels for English collocations: incongruent (e.g., “lose count”), partially congruent (e.g., “black tea”), and congruent (e.g., “good luck”). Following the criteria set previously, the research group randomly selected 16 sets of English collocations for each category (8 verb collocations and 8 noun collocations), totaling 48 sets of collocations from the corpus (see Table 4). Variance analysis showed that there was no significant difference in average word length (*F* (2.45) = 0.006; *p* > 0.05), average word frequency (*F* (2.45) = 0, *p* > 0.05), and average collocation frequency (*F* (2.45) = 2.50, *p* > 0.05) among the three levels of collocations. The congruency score of each collocation group was 1, 2, or 3: level 1 referred to “none of the component words are correspond to any component word in Chinese counterpart,” level 2 referred to “at least one component word corresponds to one component word in Chinese counterpart,” level 3 referred to “all the component words correspond to the component words in Chinese counterpart.” In order to ensure the objectivity of evaluating and distinguishing congruency, apart from three linguistic professionals, two additional linguistic experts were invited to evaluate the collocations. The finally selected experimental materials must receive congruency ratings from all five researchers.

**Table 4:** Examples of collocations in congruency range.

Congruency range	Examples	Average word frequency (SD)	Average word length (SD)	Average collocation frequency (SD)
Congruent (16)	True love, save time	38,432 (5,743)	3.56 (1.45)	145 (26.89)
Partially congruent (16)	Bank holiday, kill time	38,765 (4,364)	4.23 (1.23)	134 (28.32)
Incongruent (16)	Think tank, catch cold	38,166 (3,956)	3.78 (1.32)	153 (32.14)

The process of this experiment was similar to Experiment 1, where the 48 sets of collocations were randomly presented in a familiarity questionnaire. To avoid the cross-influence between the two experiments, Experiment 2 was conducted 2 weeks after completion of Experiment 1 questionnaire. After the participants completed the questionnaire for Experiment 2, the researchers input the variables of familiarity, congruency, and participants’ L2 proficiency into a computer and analyzed the data using SPSS 19.0 software.

### 3.3 Results

Similar to Experiment 1, the research group first analyzed the correlation between the L2 collocational familiarity of different learners and the congruency. The statistical results of Spearman correlation coefficient are shown in Table 5: There was a moderate and significant correlation between the familiarity of learners with collocations and their congruency (*r* = 0.510; *p* < 0.01) overall. Both high-level and low-level learners showed a significant correlation between familiarity and congruency, with the correlation coefficient for high-level learners being significantly lower than that of low-level learners(*Z* = 1.86, *p* < 0.05).

**Table 5:** Correlation between collocational familiarity and congruency of different L2 learners.

	Collocational familiarity		
	Overall	Low-level learners	High level learners
Congruency	*r* = 0.510	*r* = 0.675	*r* = 0.502
	Sig.(2-tailed) = 0.000	Sig.(2-tailed) = 0.000	Sig.(2-tailed) = 0.000

Subsequently, the research group conducted further comparative analysis on the familiarity data mentioned above, and the results showed (see Table 6): (1) There were significant differences in familiarity among different congruency ranges, with the order of performance being: congruent > partially congruent > incongruent. Friedman’s test indicated that this difference reached a statistically significant level (p < 0.01). (2) There were certain differences between learners of different proficiency levels. Within collocations of the same congruency level, high-level learners performed differently from low-level learners. However, Mann–Whitney’s test showed that, in the congruent range, the difference between the two levels of learners did not reach a statistically significant level (*p* > 0.05) in terms of familiarity. On the other hand, in terms of familiarity in partially congruent and incongruent collocations, the difference between the two types of learners reached a significant level (*p* < 0.01).

**Table 6:** Collocational familiarity of different learners among different congruency ranges.

	Low-level learners (SD)	High-level learners (SD)	Mann-Whitney U*	p*
Congruent	4.8285 (0.79)	4.9365 (0.41)	298	0.175
Partially congruent	3.3600 (0.73)	4.1817 (0.59)	221	0.000
Incongruent	2.8238 (0.26)	3.3290 (0.91)	384	0.000
Chi-Square**	58.985	56.613		
df**	2	2		
P**	0.000	0.000		

*Mann–Whitney Test; **Friedman Test.

### 3.4 Discussion

The results of Experiment 2 indicate that L2 familiarity and congruency are positively correlated at the intermediate level, with learners being more familiar with collocations with higher congruency. This is primarily due to the “same-translation effect” where the first language accelerates the processing of second language collocations (Yamashita and Jiang, 2010; Wolter and Gyllstad, 2011; Zhang et al., 2017). These studies demonstrated the processing advantage of congruent collocations: when learners encounter unfamiliar collocations, they tend to employ a mode of decomposition and analyze the component words. In this case, learners activate the first language semantics to process the second language semantics before entering into second language form processing. As a result, congruent collocations are more likely to be noticed, understood, and solidified in learners’ mental lexicons, while non-equivalent collocations are more likely to be ignored. Consequently, congruent collocations have a higher degree of solidification and familiarity in learners’ mental representations. However, the processing advantage of congruent collocations should not solely be attributed to the “same-translation effect.” Another plausible explanation is the Age/Order of Acquisition hypothesis. This hypothesis posts that the processing speed of earlier acquired vocabulary is faster than that of later acquired vocabulary in semantic tasks (Ellis and Lambon Ralph, 2000) because the earlier acquired vocabulary remains in a privileged position in the lexical associative network (supremacy) due to continuous learning.

On the other hand, the correlation coefficient of familiarity-congruency of high-level learners was significantly lower than that of low-level learners, and for high-level learners, familiarity with non-congruent collocations was significantly higher. This result indicates that the same-translation effect is more prominent in collocation processing for low-level learners and gradually diminishes with the frequency effect at the advanced stage. This finding complements the conclusions of Chen et al. (2016) and Wei and Zhang (2017).

## 4 General discussion

**Figure 1:**
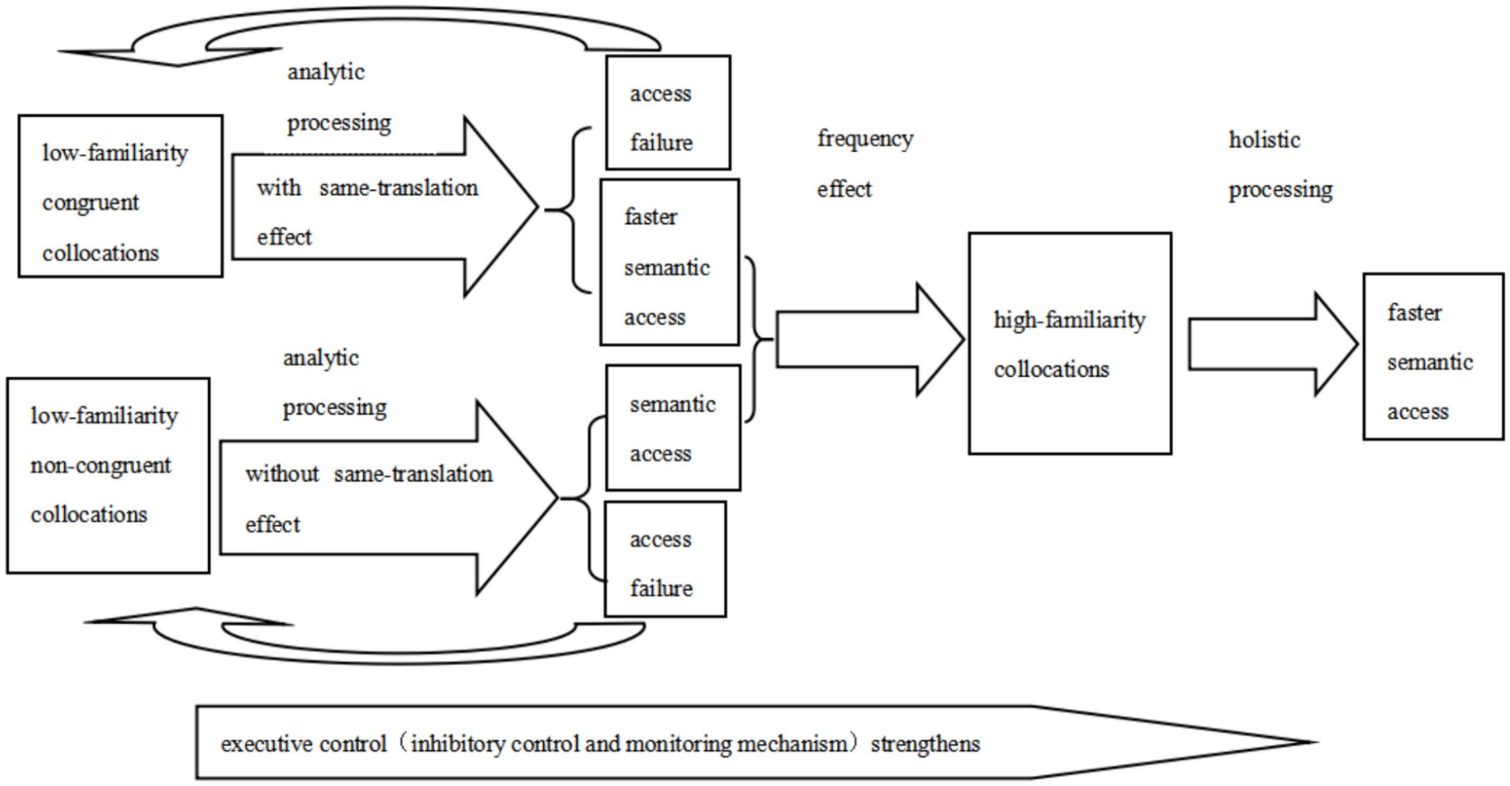
Model of L2 collocation processing and development.

From the two experiments above, the research group found that the relationship between collocational frequency, congruency and collocational familiarity varied with the development of L2 proficiency. The relationship between familiarity and frequency strengthened as proficiency increases, while the relationship between familiarity and congruency weakened with proficiency improvement. This indicates that collocational familiarity, collocational frequency, and congruency may play different roles in L2 collocation processing and development. In L2 collocational processing, frequency is the independent variable and familiarity is the moderating variable during the early stages of collocation learning. Based on Van Lancker Sidtis’s (2012) model and the findings of this study, the research group created the following model of L2 collocational processing and development (see Figure 1): low familiarity collocations tend to be processed analytically and benefit from the same-translation effect, leading to faster semantic access; low familiarity collocations gradually develop into high familiarity collocations with increased repetition frequency; high familiarity collocations achieve direct semantic access through holistic processing. The faster semantic access of high-familiarity collocations may be attributed to enhanced executive control in working memory, including inhibitory control and monitoring mechanisms, which favor holistic processing (Cai, 2015). The findings of this study also support Van Lancker Sidtis’s (2012) proposed “dual-route model” of chunks, which suggests that both holistic access and analytic decomposition coexist and compete with each other.

**Figure 2:**
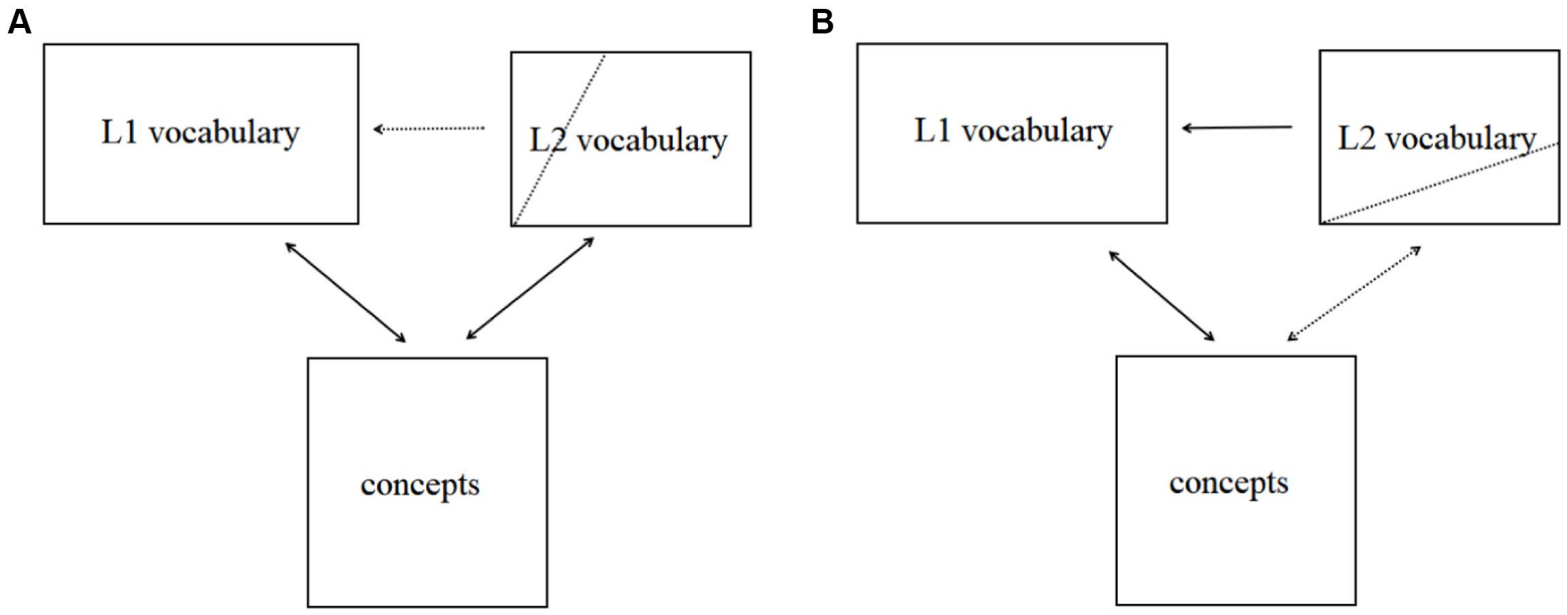
Bilingual lexical representation of different L2 learners. **(A)** Lexical representation of high-level L2 learners. **(B)** Lexical representation of low-level L2 learners.

The findings of this study refute Wray’s (2002) conclusion that L2 collocations lack holistic representation and further supplement the conclusions of Wolter and Gyllstad (2011) and Wang (2015) by suggesting that the holistic representation of L2 collocations depends on familiarity rather than the language user; high familiar collocations tend to be holistically represented, while low familiar collocations tend to be analytically represented. The representation of L2 collocations is gradually established as proficiency and familiarity with the collocations increase, and both high and low level learners possess some degree of holistic collocation representation. With the gradual establishment of L2 representation, how do bilingual speakers connect their two languages and in what ways? Currently, most scholars agree on the representation approach of “lexical separation and conceptual sharing,” with the revised hierarchical model proposed by Kroll and Stewart (1994) being the most renowned. Based on Kroll and Stewart’s (1994) revised hierarchical model and the findings of this study, the research group proposed a bilingual representation model for learners at different levels (Figure 2), suggesting that both high and low level learners have two representations and two semantic access modes for L2 collocations, with low-level learners having more unfamiliar collocations, thus exhibiting more analytical representation and indirect semantic access. These conclusions align with (Jiang, 2000) three-stage model of vocabulary development, which only described bilingual vocabulary representation from a single-word perspective, whereas this study extends the scope of description to multi-word units.

## 5 Conclusion and implications

The present study, through non-parametric correlation analysis and comparative analysis, found that under the same conditions, there was a moderate positive correlation between L2 collocational familiarity and collocational frequency, as well as congruency. Learners were more familiar with high frequency and congruent collocations. With the improvement of proficiency, the correlation between familiarity and frequency strengthened, while the correlation between familiarity and congruency weakened. Based on these findings, this study proposed L2 collocation development model and bilingual representation model of different leveled learner. However, this study adopted a cross-sectional design, and subsequent research could consider adopting longitudinal research methods to more comprehensively reveal the developmental patterns of L2 learners’ collocational familiarity. In addition, the results of this study also indicated that L2 learners’ collocation knowledge still needed to be strengthened. English vocabulary teaching should shift the focus from vocabulary breadth knowledge to vocabulary depth knowledge in order to improve collocation knowledge through intentional learning and incidental learning. However, issues such as which learning method is more effective, and which variables need to be adjusted to improve effectiveness in collocation learning, still require further research.

## Data availability statement

The raw data supporting the conclusions of this article will be made available by the author, without undue reservation.

## Ethics statement

The studies involving humans were approved by Zhejiang Shuren University Ethics Committee. The studies were conducted in accordance with the local legislation and institutional requirements. The participants provided their written informed consent to participate in this study.

## Author contributions

JL: Investigation, Writing – original draft.
