# Supplementary material for: The development of L2 collocational familiarity and its relationship with collocational frequency and congruency
Source: Front Psychol. 2024 Jul 1;15:1332692. doi: 10.3389/fpsyg.2024.1332692 (PMC11247018; doi:10.3389/fpsyg.2024.1332692)
Supplement: Supplementary file 1 [file Table_1.DOCX]

**Appendix: Collocations of Different Frequency Range**

**Low frequency： F: 21-30**  **MI≥3**

| **Collocations** | **BNC frequency** | **Log-transformed frequency** | **Collocations** | **BNC frequency** | **Log-transformed frequency** |
| --- | --- | --- | --- | --- | --- |
| reach decisions | 24 | 1.380211 | slow learner | 24 | 1.380211 |
| serve a purpose | 21 | 1.322219 | cheap way | 30 | 1.477121 |
| bite the dust | 27 | 1.431364 | white lie | 24 | 1.380211 |
| give an order | 21 | 1.322219 | famous phrase | 25 | 1.39794 |
| breathe a word | 29 | 1.462398 | dark water | 27 | 1.431364 |

**Medium-low frequency F: 31-60 MI≥3**

| **Collocations** | **BNC frequency** | **Log-transformed frequency** | **Collocations** | **BNC frequency** | **Log-transformed frequency** |
| --- | --- | --- | --- | --- | --- |
| take pains | 54 | 1.732394 | early bird | 35 | 1.544068 |
| save face | 31 | 1.491362 | hard luck | 59 | 1.770852 |
| enter service | 42 | 1.623249 | fixed sum | 38 | 1.579784 |
| carry the burden | 37 | 1.568202 | iron works | 39 | 1.591065 |
| follow instructions | 40 | 1.60206 | major success | 60 | 1.778151 |

**Medium-high frequency F: 61-100 MI≥3**

| **Collocations** | **BNC frequency** | **Log-transformed frequency** | **Collocations** | **BNC frequency** | **Log-transformed frequency** |
| --- | --- | --- | --- | --- | --- |
| catch the eye | 95 | 1.977724 | blind date | 61 | 1.78533 |
| open arms | 65 | 1.812913 | small fortune | 81 | 1.908485 |
| answer the phone | 64 | 1.80618 | sharp rise | 89 | 1.94939 |
| bear fruit | 95 | 1.977724 | stone age | 72 | 1.857332 |
| find jobs | 91 | 1.959041 | difficult problem | 76 | 1.880814 |

**High frequency F>100 MI≥3**

| **Collocations** | **BNC frequency** | **Log-transformed frequency** | **Collocations** | **BNC frequency** | **Log-transformed frequency** |
| --- | --- | --- | --- | --- | --- |
| make sense | 1700 | 3.230449 | public law | 297 | 2.472756 |
| pay attention | 368 | 2.565848 | senior citizen | 128 | 2.10721 |
| take charge | 437 | 2.640481 | body weight | 325 | 2.511883 |
| take place | 10434 | 4.018451 | flat rate | 110 | 2.041393 |
| give effect | 227 | 2.356026 | poor health | 117 | 2.068186 |
